# Efficacy of the Hong Kong‐Vigilance and Memory Test Platform on Early Detection of Cognitive Deficits and Promotion of Healthy Behaviors in Older Adults

**DOI:** 10.1111/ggi.70214

**Published:** 2025-10-14

**Authors:** Ada Wai Tung Fung, Suk Ling Ma

**Affiliations:** ^1^ Department of Sports and Health Sciences, Academy of Wellness and Human Development, Faculty of Arts and Social Sciences Hong Kong Baptist University Hong Kong China; ^2^ Life Course Development Research Institute, Faculty of Arts and Social Sciences Hong Kong Baptist University Hong Kong China; ^3^ School of Arts and Humanities Tung Wah College Hong Kong China

**Keywords:** brain health, cognitive screening, community health program, early cognitive deficits, early detection, preventive care

## Abstract

**Introduction:**

The global rise of cognitive impairment and dementia poses significant public health challenges. The Hong Kong‐Vigilance and Memory Test (HK‐VMT) platform combines dementia risk assessment and cognitive tests in one accessible tool to enable early detection of dementia in a community setting. This study aimed to evaluate the effectiveness of the HK‐VMT platform in assessing dementia risk and a broad spectrum of cognitive impairment in community‐dwelling adults. It also assesses the impact of the platform on improving public awareness and encouraging lifestyle changes.

**Methods:**

This cross‐sectional study assessed 517 adults aged 50 and above recruited through outreach activities between July 2024 and March 2025. Participants underwent a two‐stage screening process consisting of dementia risk assessment and cognitive test. The platform collected data on socio‐demographic, psychological, medical, and physiological factors for assessing dementia risk using Cognitive Ageing Risk Score (CARS). Cognitive performance was measured by the HK‐VMT. User feedback on platform accessibility, adoption, user engagement, public awareness, and attitudes toward healthy lifestyles was obtained through interview.

**Results:**

Before screening, 78% of participants with cognitive impairments were unaware of their condition. Cognitive deficits were detected in 11.4% of participants. Over 80% expressed intentions to adopt a healthy lifestyle after screening.

**Conclusion:**

The HK‐VMT platform shows enhanced early detection of cognitive impairments, improved accessibility, increased public awareness, and engaged the public in brain health management. It represents a scalable solution to support healthy aging and reduce disparities in early dementia preventive care by bridging community cognitive health services.

## 1 Background

Dementia affects 57 million people worldwide in 2021 and is projected to triple by 2050 [1]. While the prevalence of dementia increases with age, growing evidence suggests that untreated physical and mental health conditions and poor lifestyle choices are also key contributors to the rise of dementia [2, 3, 4, 5, 6]. The 2024 Lancet Commission report highlighted that nearly half of the incidence could be prevented by addressing 14 modifiable risks, including low education, hearing loss, hypertension, smoking, obesity, depression, physical inactivity, diabetes, excessive alcohol consumption, traumatic brain injury, air pollution, infrequent social contact, untreated vision loss, and elevated LDL levels [7]. It is crucial to identify individuals at risk of dementia and to promote a healthy lifestyle early. However, many community centers providing social services for older people at the district level lack the resources and expertise for comprehensive dementia evaluations, which limits timely interventions and efficient allocation of preventive care.

To address this service gap, the Hong Kong‐Vigilance and Memory Test (HK‐VMT) platform was launched in mid‐2024. The HK‐VMT platform is a web application that combines Cognitive Ageing Risk Score (CARS) and HK‐VMT to enhance dementia risk assessment and early detection of dementia at the community level. The CARS is a dementia risk assessment tool for estimating the risk of developing dementia based on key risk factors. The HK‐VMT is a simple self‐administered computerized cognitive screening tool developed in 2021 to detect early cognitive impairment. It can be completed in 15 min. The HK‐VMT was integrated with CARS in 2024 to become the current HK‐VMT platform. The development and validation of both tools have been described elsewhere [8, 9]. In brief, the platform uses dementia risk assessment to identify individuals who may need further cognitive testing. The integration has significantly increased the HK‐VMT uptake rate from 10% annually to 82% in 2024, demonstrating the effectiveness of this combined approach. The platform is currently used by 2047 users across 20 District Elderly Community Centres (DECCs) and Neighbourhood Elderly Centres (NECs).

The HK‐VMT platform is specifically designed to detect subtle cognitive deficits in the preclinical phase to facilitate early intervention. It has two separate registration portals for individual users and professionals. Individual users who register through the public portal can link their personal account with their healthcare providers. This allows authorized professionals to access and view test results of the linked users, enabling the organization to monitor cognitive status and allocate resources effectively. The platform aims to provide a free online tool for the public to detect early cognitive changes, raise public awareness of personal dementia risk factors, provide lifestyle advice to reduce dementia risks in high‐risk individuals, and support service providers in tracking the cognitive health of their clients.

The current study evaluated the effectiveness of the platform in detecting early cognitive deficits, raising public awareness of brain health, and promoting healthy behaviors. It also estimated the prevalence of dementia risk and the severity distribution of cognitive impairments using the HK‐VMT platform.

## 2 Methods

### 2.1 Study Design and Participants

This cross‐sectional study was conducted between July 2024 and March 2025; a total of 517 participants aged 50 or above were recruited and assessed at community outreach events. Data collected via the HK‐VMT platform included socio‐demographic, psychological, cognitive, medical, and physiological factors. Buccal DNA samples were collected for Apolipoprotein E (APOE) genotyping. Cognitive performance was evaluated using HK‐VMT. Research assistants administered the Hong Kong version of the Montreal Cognitive Assessment (HK‐MoCA) [10]. A user feedback survey was administered to assess platform adoption, user engagement, public awareness, and attitudes toward healthy lifestyles.

### 2.2 Description of the Two‐Stage Screening of the HK‐VMT Platform

Stage 1 Dementia risk assessment: The CARS estimates the 6‐year dementia risk based on demographic, health, and behavioral risk factors in Chinese older adults. These factors include age, sex, years of education, diabetes, engagement in mind–body exercise, physical inactivity, sleep quality, and level of loneliness [8]. A CARS cutoff of −1.3 or above has a sensitivity and specificity of 83.9% and 75.4% for predicting dementia risk. On the platform, these risk factors are assessed through a survey. Four additional dementia risk factors, including hyperlipidemia, hypertension, obesity, and smoking status, are also included to generate tailored lifestyle advice promoting cognitive and physical activity, weight management, healthy diets, and stress reduction.

Stage 2 Cognitive assessment: Individuals identified as having high risk of dementia by CARS proceed to cognitive testing using the HK‐VMT [9]. The test consists of four subtests assessing attention, psychomotor speed, visuospatial memory, learning, and episodic memory. Each subtest generates an individual score, which is summed together to form a total score ranging from 0 to 40, with higher scores indicating better cognitive performance. A cut‐off of 21/22 differentiates Mild Cognitive Impairment (MCI) from normal cognition with a sensitivity of 86.1% and specificity of 75.3% [9]. Raw scores from each subtest are standardized into age‐ and education‐adjusted *z*‐scores to calculate the global composite cognitive score.

### 2.3 Result Dashboard

Upon completion of the two‐stage screening process, an immediate comprehensive report is displayed on the result dashboard. The report informs users of their cognitive test score, dementia risk level, the likelihood of developing dementia in 6 years expressed as a percentage, attributable modifiable risk factors, and personalized lifestyle recommendations tailored to individual needs. The HK‐VMT platform classifies cognitive status into four color‐coded categories: green for cognitively normal, yellow for cognitive deficits, orange for MCI, and red for significant cognitive impairments suggestive of dementia. Users can log in to their personal accounts to access their previous test results and personalized recommendations, so as to enable tracking of their cognitive change over time.

### 2.4 Evaluation of the HK‐VMT Platform

Effectiveness in early detection of dementia was assessed by comparing the distribution identified by the HK‐VMT and the HKMoCA. This comparison quantified the platform's ability to detect previously unrecognized cognitive impairments relative to conventional screening methods. Psychosocial and behavioral outcomes were assessed by measuring public awareness of brain health, identifying barriers to test accessibility, and evaluating attitudes toward healthy lifestyle behaviors.

### 2.5 Statistical Analysis

Descriptive statistics were used to summarize participant characteristics on sociodemographic variables, dementia risk, and their cognitive profile. Difference in cognitive performance between individuals with and without dementia risk factors was analyzed using independent *t* tests. Associations between modifiable risk factors and cognitive performance were examined using univariate analysis. Changes in knowledge in cognitive health and confidence in managing brain health were analyzed using paired *t* tests. Data analyses were performed in IBM SPSS Statistics 28.0 for Windows. Statistical significance was defined as *p* < 0.05 for all analyses.

## 3 Results

### 3.1 Participant Characteristics

A total of 521 participants were initially assessed, with four participants excluded due to incomplete data. This resulted in a final sample of 517 individuals for analysis. Nearly half (48.2%) of them were recruited through DECC and NEC, while the remainder responded to public advertisements. The mean age of the entire sample was 69.3 ± 7.5 years with 80.1% female participants. Most participants (78.9%) had completed at least 7 years of completed education, corresponding to a secondary school level of education. On average, the cohort exhibited a low dementia risk with normal cognition, reflected by a mean CARS score of −3.3 ± 2.5 and a mean HK‐VMT score of 27.3 ± 5.1. Detailed demographic characteristics are summarized in Table 1.

**TABLE 1:** Demographic Characteristics of the 517 participants.

Characteristics	All (*N* = 517)		
	Mean	(SD)	%
Age	69.3	(7.5)	—
Female	—		80.1
Education			
Years	10.7	(4.5)	
Primary	—		21.1
Secondary	—		54.7
Tertiary	—		24.2
ApoE4 Genotype			
ε4 carrier	—		18.4
CARS	−3.3	(2.5)	—
HK‐VMT	27.3	(5.1)	—
HK‐MoCA	26.6	(3.6)	—

### 3.2 Prevalence of Cognitive Impairment

Cognitive impairments were identified in 34.3% (*N* = 177) participants. Specifically, 11.4% exhibited cognitive deficits, 21.9% had MCI, and 1% demonstrated significant cognitive impairments suggestive of dementia. The prevalence of cognitive impairments increased significantly with age (*r* = 56.4, *p* < 0.001). Compared to the HK‐MoCA, which identified 10.1% of participants with MCI, the HK‐VMT platform detected an additional 15.9% of MCI cases. Table 2 represents the distribution of cognitive status stratified by age groups.

**TABLE 2:** Prevalence of cognitive impairments (in %) classified by HK‐VMT.

Age	CN (*N* = 340)	CD (*N* = 59)	MCI (*N* = 113)	SCI (*N* = 5)
50–59 years	84.4 (27)	6.3 (2)	9.4 (3)	0
60–69 years	76.1 (197)	9.7 (25)	14.3 (37)	0
70–79 years	56.6 (99)	14.3 (25)	27.4 (48)	1.7 (3)
80 years	33.3 (17)	13.7 (7)	49 (25)	3.9 (2)

Abbreviations: CD, cognitive deficit; CN, cognitively normal; MCI, mild cognitive impairment; SCI, significant cognitive impairment.

A one‐way ANOVA revealed significant differences in HK‐VMT total scores (*F* = 225.9, *p* < 0.001, *η* ^2^ = 0.57) across the four cognitive statuses. Differences were also observed in immediate recall (*F* = 104.0, *p* < 0.001, *η* ^2^ = 0.38), delayed recall (*F* = 126.5, *p* < 0.001, *η* ^2^ = 0.43), attention (*F* = 10.0, *p* < 0.001, *η* ^2^ = 0.06), and visuospatial memory (*F* = 62.3, *p* < 0.001, *η* ^2^ = 0.3).

### 3.3 Prevalence of Dementia Risk Factors and Associations With Cognition

Nearly all participants (98%) presented with at least one modifiable risk factor. Based on CARS, 19.7% were classified as high risk for developing dementia. The most prevalent risk factors were poor sleep quality (67.7%), loneliness (65.2%), and hypertension (50.1%). Other notable risk factors were hyperlipidemia (23%), diabetes (13.5%), overweight or obesity (10.1%), and smoking (0.4%). While the cohort was generally physically active, with only 4.8% having physical inactivity, nearly half (48.9%) did not engage in mind–body exercises.

Univariate analyses examined the relationship between individual modifiable risk factors and cognitive performance. Table 3 displayed the difference in HK‐VMT total score between those with and without an individual risk factor. Participants with diabetes, hypertension, poor sleep, loneliness, or lack of mind–body exercise demonstrated significantly lower HK‐VMT scores compared to those without these risk factors. Further analysis explored whether carrying the ApoE ε4 allele modified the association between modifiable risk factors and cognition. A significant interaction was found between ApoE ε4 status and hypertension on cognitive performance (*F*(5, 449) = 3.09, *p* = 0.047), indicating that genetic predisposition may influence the impact of hypertension on cognitive outcomes.

**TABLE 3:** Comparison of HK‐VMT score in individuals with and without dementia risk factors.

Risk factors	Presence		Absence		*T* test
	Mean	SD	Mean	SD	*p*
Diabetes	25.2	5.4	27.6	5.0	***
Hypertension	27.3	4.9	28.6	4.5	**
Hyperlipidemia	28.3	4.9	28.2	4.9	ns
No Mindbody Exercise	26.6	5.0	27.9	5.2	**
Physical inactivity	27.8	5.4	27.2	5.1	ns
Overweight/Obesity	27.7	6.2	27.3	5.0	ns
Poor sleep	26.9	5.2	28.0	4.9	**
Loneliness	27.0	5.2	27.9	5.0	*

*Note:* ***p < 0.001; **p < 0.01, and *p < 0.05.

Abbreviations: ApoE4, apolipoprotein e4; ns, not significant; S.D., standard deviation.

### 3.4 Impact on Awareness and Motivation for Behavioral Change

Among participants identified with cognitive impairments, 78% (*N* = 138) were previously unaware of their condition. A paired samples *t* test was performed to evaluate whether there was a difference in the knowledge and confidence levels before and after screening. There was a significant difference in the knowledge scores (before 2.6 ± 0.8 and after 3.5 ± 0.8; *t* = −20.8, *p* < 0.001). Similarly, there was a significant difference in the confidence levels (before 3.1 ± 0.9 and after 3.7 ± 0.8; *t* = −13.9, *p* < 0.001).

A substantial proportion of participants with cognitive impairments expressed intentions to adopt a healthier lifestyle, with 87% planning to increase their physical activity, 77% intending to improve their diet, and 70% aiming to pay closer attention to brain health information; 58% considered undergoing regular checkups on their cardiovascular health; and 54% planned to engage more in volunteering or social activities. Qualitative feedback from participants highlighted increased awareness, motivation for lifestyle changes, and a desire for ongoing cognitive health monitoring. For example, participants noted:

- “Very helpful, especially for community older people who generally have limited access to such information.” (BHD480)
- “The screening helped me to gain a deeper understanding of the knowledge about memory and alertness. I can also refer to the report to improve my poor habits.” (BHD403)
- “Thank you so much for conducting this assessment on me! It has given me a much deeper understanding of cognitive impairment, which will be incredibly helpful for my future lifestyle and healthy living habits.” (BHD429)
- “It's really great! It allows for an initial assessment of my cognitive abilities and health, which helps me to make improvement. I hope it can be done regularly, like once a year, for screening purposes.” (BHD414)

## 4 Discussion

Currently, clinical care is mainly focused on diagnosis and management after onset. The community services and supports for older people with subtle impairments remain insufficient. These social services often lack the capacity to differentiate early cognitive deficits from normal aging for timely management. The HK‐VMT platform addresses an important service gap by providing a fast and cost‐effective method to improve early detection of cognitive deficits in a community setting. The data‐driven approach analyzes behavioral patterns with health metrics to reduce over‐reliance on center staff for routine screening, while empowering them to intervene proactively in at‐risk populations.

Our study showed that over one‐third of older adults experienced varying levels of cognitive impairment in Hong Kong. Of these, 21.9% were classified as having MCI. This prevalence aligns closely with previous epidemiological estimates of 21.2%, demonstrating the validity of the platform in detecting MCI in the local community [11]. Notably, the HK‐VMT platform increased the detection rate of MCI by 15.9% compared to conventional screening. Given that over 80% of the undetected MCI cases had attained secondary education or above, the discrepancy in MCI detection suggests that traditional screening tools may be less sensitive and are more likely to underestimate the burden of early cognitive impairment in individuals with higher education levels.

As reported, over 10% of participants exhibited cognitive deficits co‐occurring with multiple modifiable risk factors such as hypertension, diabetes, poor sleep, loneliness, and lack of mind‐body exercise. The findings highlighted the heterogeneity within high‐risk groups and the need for tailored risk‐reduction strategies. Furthermore, our study found that genetic predisposition modifies the relationship between hypertension and cognitive outcomes. This suggests that early blood pressure control is important for individuals with a family history of dementia, further emphasizing the need for personalized interventions targeting specific risk factors to prevent or delay cognitive decline and dementia.

The platform also provides domain‐specific cognitive profile analysis to help identify areas where users need improvement. The two‐stage screening process enables a nuanced stratification of dementia risk to assist visualization of individual cognitive domains with reference to a predefined threshold. The findings align well with previous work showing early declines in attention and learning in individuals with cognitive deficits and a cluster of vascular, behavioral, and psychosocial risk factors [7, 8, 12, 13, 14, 15, 16, 17]. The analysis could guide the selection of domain‐specific interventions, such as adaptive cognitive training [18] or mind‐body exercises like Tai Chi, Qigong, and yoga [19]. The information could shift dementia prevention strategies from clinical care to pre‐symptomatic risk management.

Our study also showed that 80% of individuals who were previously unaware of their cognitive status reported increased intentions to adopt healthier behaviors after using the platform. The result underscores the significant impact of the HK‐VMT platform in enhancing brain health awareness and motivates proactive self‐management among older adults, potentially delaying or preventing progression to dementia. Future research should explore the long‐term adherence to these intentions to adopt a healthy lifestyle and evaluate the direct impact of the platform on cognitive outcomes over time.

This study had several limitations. First, the cross‐sectional design limits estimation of the progression rate of dementia risk and the incidence of dementia. Second, the majorities of participants were females and had higher education levels, which may limit the generalizability of our findings to males and those with lower education attainment locally and internationally. Nevertheless, the HK‐VMT platform provides valuable baseline data for longitudinal tracking of community brain health.

Our study demonstrates the value of the HK‐VMT platform in empowering users through enhanced knowledge and encouraging lifestyle modifications that may improve cognitive outcomes over time. Its integration into community health services has the potential to transform early dementia risk detection and prevention on a global scale. The initiative exemplifies a scalable approach to reduce disparities in preventive cognitive care.

## Author Contributions

Ada Wai Tung Fung performed the study, analyzed the data, and wrote the manuscript. Suk Ling Ma was responsible for genotyping analysis and contributed to the final version of the manuscript.

## Ethics Statement

The Hong Kong Baptist University Research Ethics Committee (HKBU REC/23–24/0497) approved the study protocol.

## Consent

All participants provided informed consent prior to assessment.

## Conflicts of Interest

The authors declare no conflicts of interest.

## Data Availability

Research data are not shared.
